# GH18 endo-β-*N*-acetylglucosaminidases use distinct mechanisms to process hybrid-type *N*-linked glycans

**DOI:** 10.1016/j.jbc.2021.101011

**Published:** 2021-07-26

**Authors:** Beatriz Trastoy, Jonathan J. Du, Chao Li, Mikel García-Alija, Erik H. Klontz, Blaine R. Roberts, Thomas C. Donahue, Lai-Xi Wang, Eric J. Sundberg, Marcelo E. Guerin

**Affiliations:** 1 Structural Glycobiology Lab, Structural Biology Unit, Center for Cooperative Research in Biosciences (CIC bioGUNE), Basque Research and Technology Alliance (BRTA), Bizkaia, Derio, Spain; 2 Structural Glycobiology Lab, IIS-Biocruces Bizkaia, Barakaldo, Bizkaia, Spain; 3 Department of Biochemistry, Emory University School of Medicine, Atlanta, Georgia, USA; 4 Department of Chemistry and Biochemistry, University of Maryland, College Park, Maryland, USA; 5 Institute of Human Virology, University of Maryland School of Medicine, Baltimore, Maryland, USA; 6 Department of Microbiology and Immunology, University of Maryland School of Medicine, Baltimore, Maryland, USA; 7 Ikerbasque, Basque Foundation for Science, Bilbao, Spain

**Keywords:** endo-β-*N*-acetylglucosaminidases, glycoside hydrolases, glycoprotein bioengineering, antibody glycoengineering, enzyme specificity, carbohydrate active enzymes, gut microbiome, CT-type, complex type, ENGase, endo-β-*N*-acetylglucosaminidase, ESI-MS, electrospray ionization mass spectrometry, HM-type, high-mannose type, Hy-type, hybrid type

## Abstract

*N*-glycosylation is one of the most abundant posttranslational modifications of proteins, essential for many physiological processes, including protein folding, protein stability, oligomerization and aggregation, and molecular recognition events. Defects in the *N*-glycosylation pathway cause diseases that are classified as congenital disorders of glycosylation. The ability to manipulate protein *N*-glycosylation is critical not only to our fundamental understanding of biology but also for the development of new drugs for a wide range of human diseases. Chemoenzymatic synthesis using engineered endo-β-*N*-acetylglucosaminidases (ENGases) has been used extensively to modulate the chemistry of *N*-glycosylated proteins. However, defining the molecular mechanisms by which ENGases specifically recognize and process *N*-glycans remains a major challenge. Here we present the X-ray crystal structure of the ENGase EndoBT-3987 from *Bacteroides thetaiotaomicron* in complex with a hybrid-type glycan product. In combination with alanine scanning mutagenesis, molecular docking calculations and enzymatic activity measurements conducted on a chemically engineered monoclonal antibody substrate unveil two mechanisms for hybrid-type recognition and processing by paradigmatic ENGases. Altogether, the experimental data provide pivotal insight into the molecular mechanism of substrate recognition and specificity for GH18 ENGases and further advance our understanding of chemoenzymatic synthesis and remodeling of homogeneous *N*-glycan glycoproteins.

*N*-glycosylation occurs in about 70% of secreted and membrane-bound proteins, generating remarkable structural diversity in biological systems (1). *N*-glycosylation is initiated in the endoplasmic reticulum by the “*en bloc*” transfer of a common oligosaccharide Glc_3_Man_9_GlcNAc_2_ from a dolichol-pyrophosphate derivative to specific Asn residues in nascent polypeptide chains (1). The presence of the glycosylation consensus sequence (Asn-X-Ser/Thr, where X may be any amino acid except for Pro) is necessary but not sufficient for glycosylation. Processing of Glc_3_Man_9_GlcNAc_2_ is initiated immediately after transfer, by the action of glucosidases and mannosidases (1, 2). The extreme structural diversity found in oligosaccharides linked to Asn residues in mature, fully processed glycoproteins of different organisms or of different cell types belonging to multicellular organisms originates by differential processing reactions in the Golgi apparatus (3, 4). Defects in the genes encoding proteins required for *N*-glycan biosynthesis, transport, and processing cause a large and rapidly expanding group of rare diseases known as congenital disorders of glycosylation. Since their first clinical description in 1980, 130 types of congenital disorders of glycosylation have been identified, and that number keeps rising (5, 6, 7).

All *N*-glycans share the same core sugar sequence, Manα1–6(Manα1–3)Manβ1–4GlcNAcβ1–4GlcNAcβ1. However, the composition and number of antennae that decorate *N*-glycans are highly variable. *N*-glycans in eukaryotes can be classified into three major types based on the substitution of the α(1,3) and α(1,6) antennae: complex type (CT-type), high-mannose type (HM-type), and hybrid type (Hy-type) (1). The Hy-type *N*-glycan has a dual nature: its α(1,3) antenna is equivalent to the α(1,3) antenna of the CT-type *N*-glycans, while its α(1,6) antenna is covered into the α(1,6) antenna of HM-type *N*-glycans. *N*-linked glycans markedly affect not only the native biological structure but also the function of proteins (8), modulating a wide range of molecular recognition events, including intracellular trafficking, cell adhesion, host pathogen interactions, and immune responses (9).

Chemoenzymatic synthesis using endo-β-*N*-acetylglucosaminidases (ENGases) (EC 3.2.1.96) is an approach to obtain specific and homogenous *N*-glycan chemistries on glycoproteins. ENGases are endoglycosidases that hydrolyze the β(1,4) linkage between the first two GlcNAc residues of *N*-linked glycans on proteins. According to the Carbohydrate-Active Enzymes database (CAZy; www.cazy.org) (10), ENGases are classified into two families (11): GH18 and GH85. ENGases follow a substrate-assisted hydrolysis mechanism with retention of the anomeric configuration (12, 13, 14, 15, 16). An aspartic acid (GH18 family members) or asparagine (GH85 family members) residue stabilizes the reaction intermediate by hydrogen bonding interaction and orients the oxygen of the 2-acetamide group of GlcNAc (−1) to attack the anomeric carbon and form the oxazoline intermediate. A glutamic acid acts as an acid in this first step protonating the anomeric carbon and as a base in a second step deprotonating a water molecule that produces the second nucleophilic attack. These enzymes, when in the presence of the two products of hydrolysis, the hydrolyzed *N*-glycan and the protein bearing a GlcNAc residue, are also capable of catalyzing the reverse reaction toward the glycosidic bond formation. The efficiency of this reaction is increased using a glycosynthase ENGase mutant (17) and a *N*-glycan oxazoline as an activated glycosyl donor substrate (Fig. 1) (18, 19, 20, 21). In the glycosynthase mutants, the aspartic/asparagine residue that promotes the formation of the reaction intermediate is mutated in such a way that the enzyme does not catalyze the hydrolysis reaction but instead uses the synthetic glycan oxazoline, a mimetic of the reaction intermediate, for the glycosylation of an acceptor (22). The *in vitro* remodeling of the *N*-glycan attached to the crystallizable fragment region (Fc region) of immunoglobulin G (IgG) monoclonal antibodies exemplifies the application of the dual glycoside hydrolase and glycosynthase activities of ENGases (22, 23). IgG monoclonal antibodies are a prominent and expanding class of therapeutics used for the treatment of diverse human disorders including cancer, autoimmunity, and neurodegenerative and infectious diseases. IgG activities, as mediated by their effector functions, depend on the chemistry of their core *N*-glycans linked to Asn297 (24, 25, 26, 27, 28). Several studies have demonstrated that the carbohydrate composition of this *N*-glycan critically affects the interaction with the Fc-receptor and the complement system, mediating the effector functions of IgGs (29, 30). Thus, glycoengineering of this *N*-glycan on therapeutic IgGs has been developed as a strategy to produce IgGs with improved therapeutic properties (31, 32, 33). Moreover, the tumor necrosis factor alpha (TNFα) is a cytokine that plays an important role in the immune system, modulating multiple cell-signaling pathways. Recent experimental evidence indicates that TNFα increases HM-type and Hy-type *N*-glycans expression at endothelial cells and these *N*-glycans are important in regulating monocyte trafficking by mediating rolling and adhesion (34). However, understanding the biological function of glycoproteins bearing Hy-type *N*-glycans remains an intriguing and major challenge.

**Figure 1:**
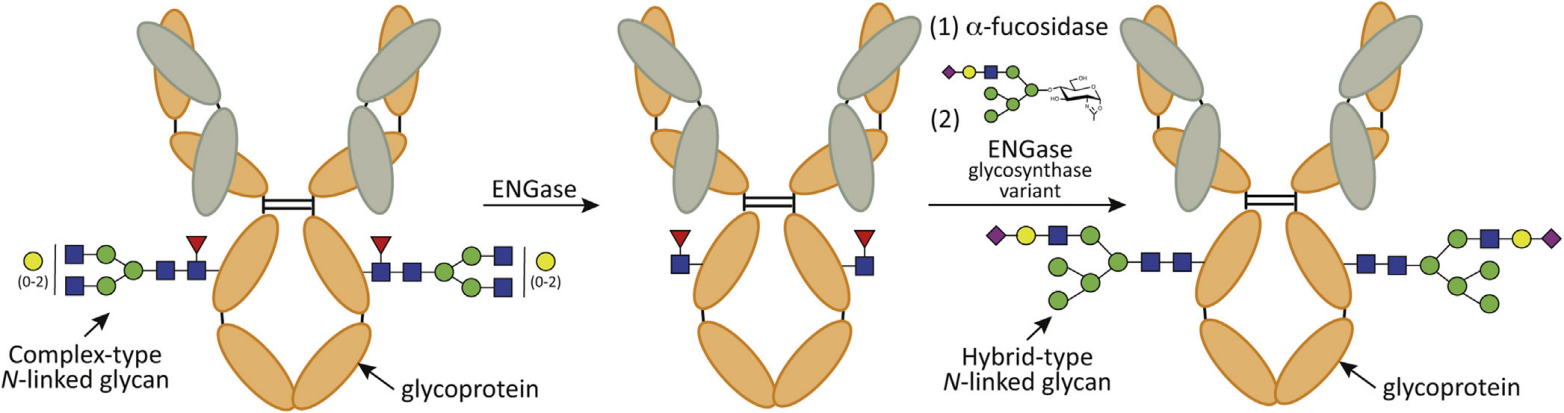
**Glycoengineering of *N*-glycan glycoproteins.** Wildtype ENGase (*e.g.*, EndoS2) specifically hydrolyzes the *N*-linked glycan on the Asn297 of the Fc region of IgG antibodies. ENGase glycosynthase mutant (*e.g.*, EndoS2_D184M_) efficiently transfers hybrid-type *N*-glycan from Hy-type oxazoline donor substrates.

Within the GH18 ENGase family, there are enzymes that play critical biological roles in a broad range of organisms, ranging from bacteria, through fungi, to higher-order species, including humans. Endoglycosidase S (EndoS, 108 kDa) and Endoglycosidase S2 (EndoS2, 92 KDa) from the obligate human pathogen *Streptococcus pyogenes* are the only enzymes with a known specific protein substrate, the Fc region of IgG antibodies. The enzymes allow the bacterium to remove more than 20 glycoforms from antibodies, eliminating their effector functions to evade the immune system. All other known ENGases can process either the *N*-glycan of IgG or those of other glycoproteins (35). Although both family of enzymes, GH18 and GH85, share common glycoside hydrolase (12, 13, 14, 15, 16) and glycosynthase catalytic mechanisms, they display different *N*-glycan specificities. EndoS is highly restrictive, only capable of hydrolyzing biantennary CT *N*-glycans, whereas EndoS2 has a much broader glycan substrate specificity—it is able to hydrolyze the three major *N*-glycans in eukaryotic cells. Conversely, EndoF1 from *Elizabethkingia meningoseptica* and EndoH from *Streptococcus plicatus*, an enzyme extensively used in biotechnology, hydrolyze HM-type and Hy-type *N*-glycans but cannot process CT *N*-glycans. To date, no enzyme has been described to exclusively hydrolyze only HM-type or Hy-type *N*-glycans.

In this work, we aim to understand the molecular mechanism by which paradigmatic ENGases are able to recognize Hy-type *N*-glycans and thus remodel glycoproteins. To this end, we focus on two GH18 enzymes, EndoS2 from *S. pyogenes* and EndoBT-3987 from *Bacteroides thetaiotaomicron*, a key enzyme that catalyzes the first step in the degradation and processing of mammalian HM-type *N*-glycans in the human gut (36, 37, 38). Altogether, our experimental data generated by the combined use of chemoenzymatic synthesis, X-ray crystallography, molecular docking calculations, and enzymatic activity measurements support a model in which EndoS2 and EndoBT-3987 use distinct molecular mechanisms to process Hy-type *N*-linked glycans.

## Results

### The crystal structure of EndoBT-3987 in complex with a Hy-type glycan product

To determine whether EndoBT-3987 is able to process Hy-type *N*-glycans we performed hydrolytic activity experiments using a chemically engineered therapeutic monoclonal antibody, rituximab, bearing Hy-type *N*-glycans (Hy-rituximab), as substrate. The Hy-rituximab was prepared *via* an endoglycosidase-catalyzed transglycosylation strategy using a Hy-type *N*-glycan oxazoline as the donor substrate. We observed that EndoBT-3987 hydrolyzes Hy-type *N*-glycans less efficiently than HM-type *N*-glycans (see Experimental procedures and ref. (31)).

**Figure 2:**
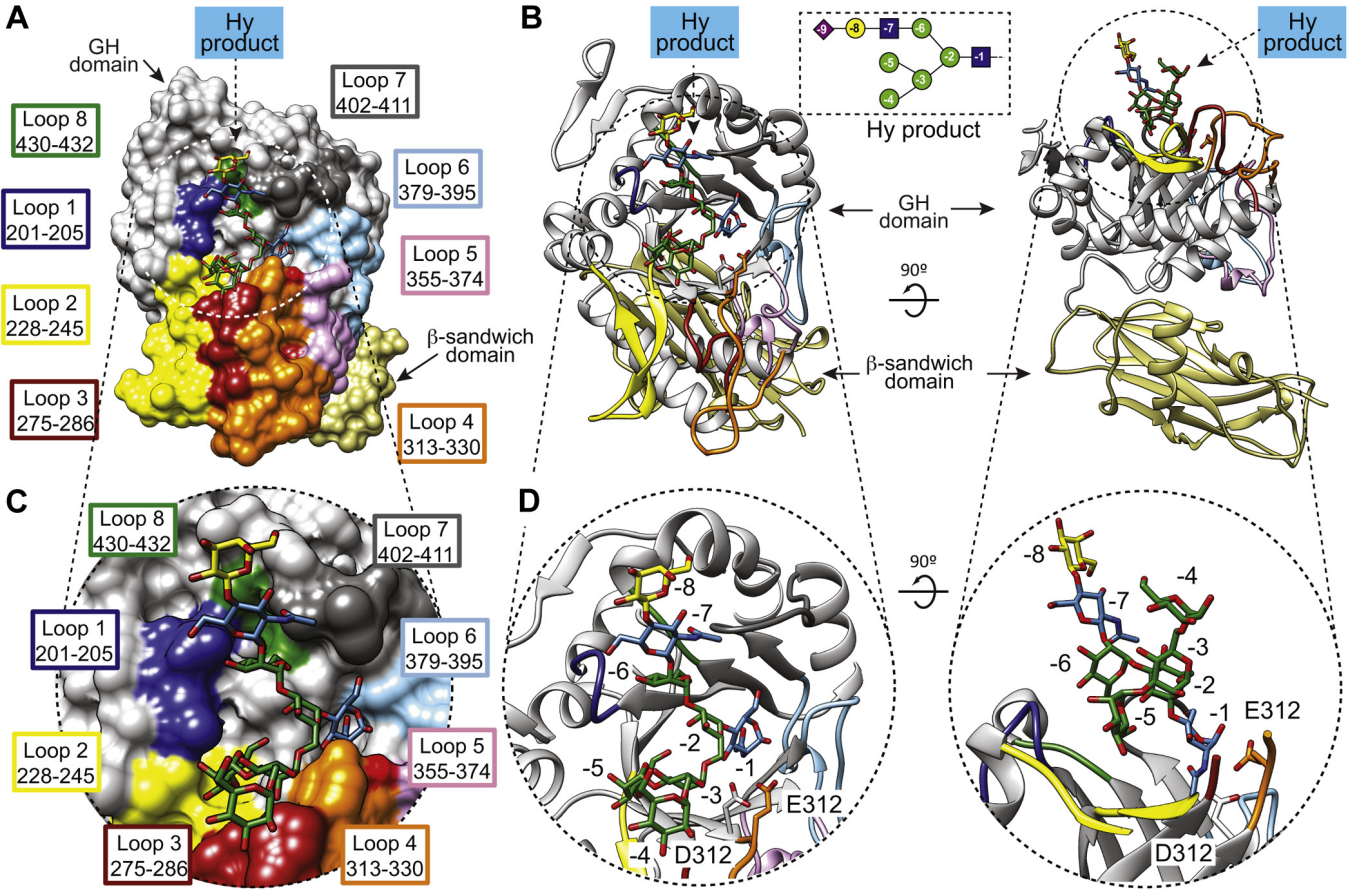
**The crystal structure of EndoBT-3987 in complex with the GalGlcNAcMan**_**5**_**GlcNAc Hy-type glycan product.***A*, surface representations of the overall structure of EndoBT-3987_WT_ in complex with a Hy-type *N*-glycan product. The glycoside hydrolase loops that decorate the Hy-type *N*-glycan product-binding site are labeled in colors. *B*, *cartoon* representation of two views of the EndoBT-GalGlcNAcMan_5_GlcNAc crystal structure. *C*, surface representation showing a close view of the Hy-type *N*-glycan product-binding site displaying the GalGlcNAcMan_5_GlcNAc molecule. *D*, *cartoon* representations showing two close views of the Hy-type *N*-glycan product-binding site displaying the GalGlcNAcMan_5_GlcNAc molecule.

**Figure 3:**
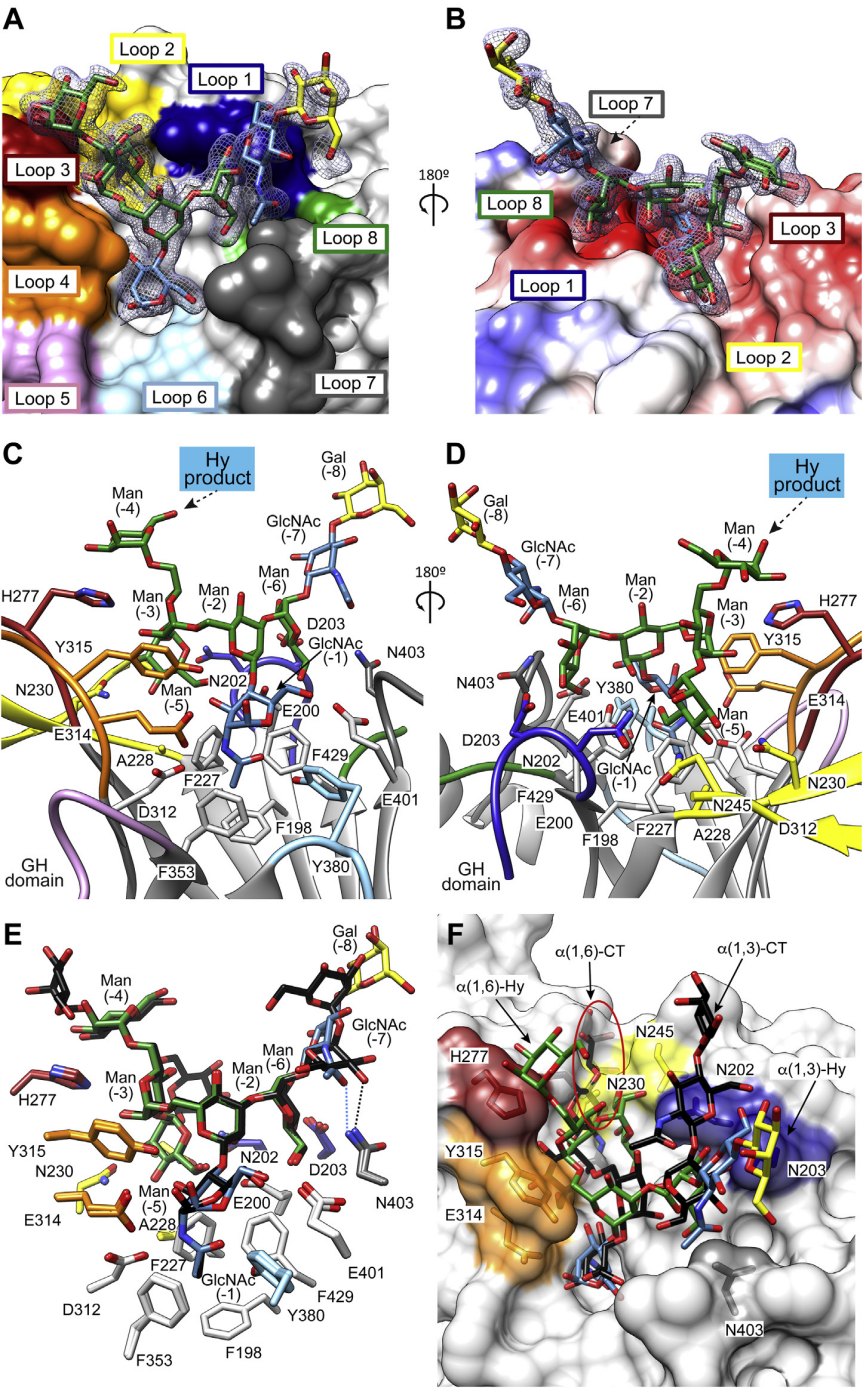
**The GalGlcNAcMan**_**5**_**GlcNAc Hy-type glycan product-binding site of EndoBT-3987.** Final electron density maps. *A* and *B*, two views of the final electron density maps (2mFo-DFc contoured at 1 σ), corresponding to the Hy-type *N*-glycan product GalGlcNAcMan_5_GlcNAc. Coulombic surface representation of EndoBT-3987 (*B*). The polar character of the enzyme is shown in three colors: negatively charged patches in red (−10 kcal mol^−1^), hydrophobic zones in white (0 kcal mol^−1^), and positively charged residues in blue (10 kcal mol^−1^). *C* and *D*, *cartoon* representation showing the loops that decorate the Hy-type *N*-glycan product-binding site. Key residues of EndoBT-3987 interacting with the Hy-type *N*-glycan product are colored. *E*, structural comparison of the EndoBT-3987_WT_-Hy and EndoBT-3987_WT_-Man_9_GlcNAc_1_ X-ray crystal structures complexes. The residues are colored by loop, and the GalGlcNAcMan_5_GlcNAc and Man_9_GlcNAc oligosaccharides are shown in different colors or *black*, respectively. *F*, superposition of EndoBT-3987-Hy (PDB code 7NWF) and EndoS2-CT (PDB code 6MDS) crystal structures. Surface representation of EndoBT-2987-Hy and the CT-type product found in the EndoS2-CT crystal structure, the key residues that interact with the Hy-type glycan are colored.

Furthermore, we determined the X-ray crystal structure of EndoBT-3987 in complex with the Hy-type *N*-glycan product Manα1,6(Manα1,3)Manα1,6(Neu5Acα2,6Galβ1,4GlcNAcβ1,2Manα1,3)Manβ1,4GlcNAc (Neu5AcGalGlcNAcMan5GlcNAc) (EndoBT-3987_WT_-Hy hereafter; Figs. 2 and 3; Table S1; Fig. S1 and Experimental procedures section; PDB code 7NWF) in the *P*2_1_2_1_2_1_ space group with one molecule in the asymmetric unit at 2.0-Å resolution (Fig. 2, Fig. S1 and Table S1) by molecular replacement using the structure of EndoBT-3987 unliganded (PDB code 3POH) as template (39). EndoBT-3987 is composed of two domains: the N-terminal β-sandwich domain (residues 42–179) and the C-terminal glycoside hydrolase domain, which adopts an (α/β)_8_-barrel topology typical of GH18 family enzymes (residues 194–476). We unambiguously identified the Hy-type *N*-glycan product in the electron density map, located at the center of the glycoside hydrolase domain in a shallow pocket decorated by the connecting loops β10–α2 (loop 1; residues 201–205), β11–α3 (loop 2; residues 228–245), β14–α4 (loop 3; residues 275–286), β15–α5 (loop 4; residues 313–330), β16–β17 (loop 5; residues 355–374), β17–β18 (loop 6; residues 379–395), β18–α6 (loop 7; residues 402–411), and β19–α7 (loop 8; residues 430–432; Fig. 2). The carbohydrate residues are numbered based on the sugar-binding subsites in glycoside hydrolases (40). Subsites are labeled from −*n* to +*n* (where *n* is an integral number); −*n* indicates the nonreducing end and +*n* the reducing end of the *N*-glycan. The hydrolysis takes place between −1 and +1 subsites. We could not reliably model the terminal Neu5Ac (−9), suggesting that this residue is solvent exposed and adopts multiple conformations in the crystal (Fig. 3, *A* and *B*). EndoBT-3897 follows a substrate-assisted mechanism with retention of the anomeric configuration, proposed for ENGases (12, 13, 14, 15, 16) (Fig. S2). D312 stabilizes the reaction intermediate by hydrogen bond interaction and orients the oxygen of the 2-acetamide group of GlcNAc (−1) in order to attack the anomeric carbon, forming the oxazoline intermediate, whereas E314 acts as an acid/base, protonating the anomeric carbon in this first step and in a second step deprotonating a water molecule that produces the second nucleophilic attack (Fig. S2). In the EndoBT-3987_WT_-Hy product crystal structure, all the sugar residues are in the energetically favorable chair conformation ^4^*C*_1_ but GlcNAc (−1) residue is in skew boat conformation (^1^*S*_3_) stabilized by interactions with protein residues. The nitrogen and oxygen atoms of the acetamide group of GlcNAc (−1) make hydrogen bonds with the side chains of Y380 and D312, respectively. The O3 and O6 of GlcNAc (−1) form hydrogen bonds with the side chains of Y315 and E401, respectively (Fig. 3, *C* and *D*). The skew boat conformation (^1^*S*_3_) is similar to that observed in other product–enzyme complex crystal structures of GH18 enzymes (39, 41, 42). The base of the binding site is mainly composed of a set of aromatic hydrophobic residues, including F198, F227, F353, and F429, located at the top of the corresponding β10, β11, β16, and β19 β-strands of the barrel, respectively. In addition, O2 of Man (−2) makes a hydrogen bond with the side chain of E200. The α(1,6) antenna (Manα1–6(Manα1–3)Manα1–6) interacts with loops 1, 2, 3, and 4, whereas the α(1,3) antenna (Galβ1–4GlcNAcβ1–2Manα1–3) is largely solvent exposed and interacts with loops 1 and 7 of EndoBT-3987, mainly through Man (−6) and GlcNAc (−7) residues (Fig. 3, *C* and *D*). The O2 of Man (−3), which is connected to Man (−4) and Man (−5), makes hydrogen bond with the side chain of H277. Moreover, Man (−3) and Man (−4) are at van der Waals contact distance of the side chains of Y315 and H277, respectively. Man (−5) is completely buried into the binding site (Fig. 3, *C* and *D*). The O2 and O6 atoms of Man (−5) make hydrogen bonds with the side chains of N202 and E200, respectively. In addition, the O3 of Man (−5) interacts with the side chains of N230 and N245 of the β-hairpin (loop2) by hydrogens bonds, whereas the O4 of Man (−5) forms hydrogen bonds with the side chain of N230 and the main chain of A229. The O4 and O6 atoms of Man (−6) of the α(1,3) antenna makes hydrogen bonds with the side chain of D203 and E200, respectively, while the oxygen of the acetamide group of GlcNAc (−7) of the same antenna interacts with the side chain of N403 through a hydrogen bond.

The structural comparison between the EndoBT-3987_WT_-Hy and EndoBT-3987_WT_-Man_9_GlcNAc (PDB code 6T8K) complexes revealed that the overall protein structure is essentially preserved (r.m.s.d. of 0.32 Å for 432 residues; Fig. 3*E*). Furthermore, residues of the loops that interact with the *N*-glycan structure also adopt a similar conformation, suggesting a common interaction mode. Incidentally, N403 shows the same conformation in the three crystal structures but this residue is able to interact by hydrogen bonds with the oxygen of the acetamide group of GlcNAc (−7) in the EndoBT-3987_WT_-Hy crystal structure and with the equivalent O4 of Man (−9) in the EndoBT-3987_WT_-Man_9_GlcNAc crystal structure (Fig. 3*E*). Although the binding pocket of EndoBT-3987 is shallow, the superposition of the EndoS2-CT (PDB code 6MDS) and the EndoBT-3987-Hy crystal structures indicates that the α(1,6) antenna of the CT glycan makes important clashes with loops 2, 3, and 4 of the protein (Fig. 3*F*). In addition, the α(1,6) antenna of CT glycans has a different chemical structure to that of HM-type glycans, lacking the bisection and Man (−3) and Man (−5) residues that stabilize the Hy-type/HM-type substrates in the active site of EndoBT-3987.

### Hy-type N-glycan recognition by EndoS2

**Figure 4:**
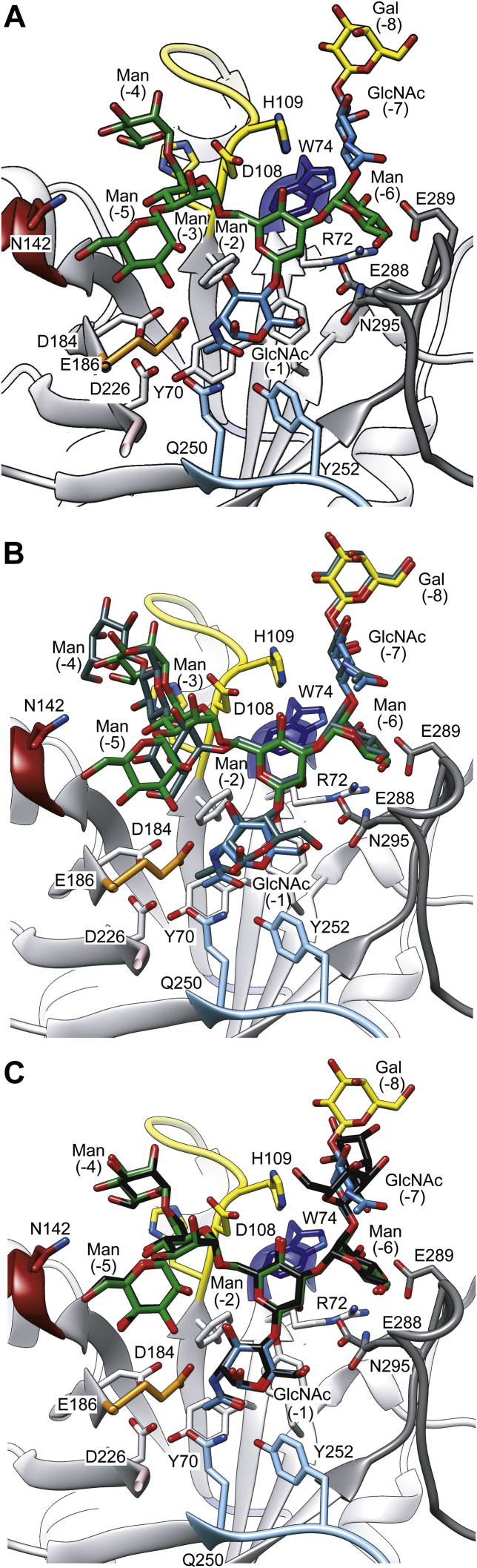
**The GalGlcNAcMan**_**5**_**GlcNAc Hy-type glycan product-binding site of EndoS2.***A*, structural comparison of the molecular docking calculations of Hy-type glycan into the active site of EndoS2 and the crystal structure of EndoS2-CT (*B*) and EndoS-HM (*C*). The CT and HM glycans are colored in *gray* and *black*, respectively.

Despite much effort, we were unable to crystallize EndoS2 in complex with the Hy-type *N*-glycan. To describe the architecture of the EndoS2 Hy-type glycan-binding site, we thus generated a three-dimensional model of the EndoS2-Hy-type *N*-glycan product complex by *in silico* molecular docking approach (Fig. 4*A*). The core Manβ1–4GlcNAc disaccharide and the α(1–3) antenna of the Hy-type glycan were placed in a similar position to those occupied in the EndoS2-CT complex structure (r.m.s.d. of 0.5 Å; PDB code: 6MDS) (Fig. 4*B*), whereas the α(1,6) antenna of the Hy-type glycan overlaps with the EndoS2-HM complex structure (r.m.s.d. of 0.5 Å; PDB code: 6MDV; Fig. 4*C*). GlcNAc (−1) and Man (−2) of the *N*-glycan core make hydrophobic interactions with residues that form the base of the binding site, including Y70, R72, F106, and Y339. The O1 atom of the first GlcNAc (−1) residue interacts with the side chain of E186, whereas O6 interacts with the side chain of N295; the oxygen and the nitrogen of the acetamide group interact with the side chain of Y252 and D184, respectively. The O2 atom of the Man (−2) residue interacts with the side chains of E288 and Y339. The carbohydrate moieties of the α(1,6) antenna barely make contacts with residues in the loops that decorate the binding pocket, in contrast with those of the α(1,3) antenna. Specifically, in the antenna α(1,3), the O3 atom of the Man (−6) residue interacts with the side chains of R72, W74, and E289; the O4 atom makes hydrogen bond with the side chain of E289, and the O6 atom interacts with the side chain of N295. The GlcNAc (−7) makes a stacking interaction with the side chain of H109, whereas Gal (−8) is exposed to the solvent. In the antenna α(1,6) the O3 atom of the Man (−5) residue interacts with the side chain of E186, whereas the O6 atom interacts with the side chains of N142, E188, and R193. The Man (−4) residue is mainly exposed to the solvent, with the O6 atom making interaction with the side chain of H107.

### Structural basis of EndoBT-3987 and EndoS2 specificity for Hy-type N-glycans

**Figure 5:**
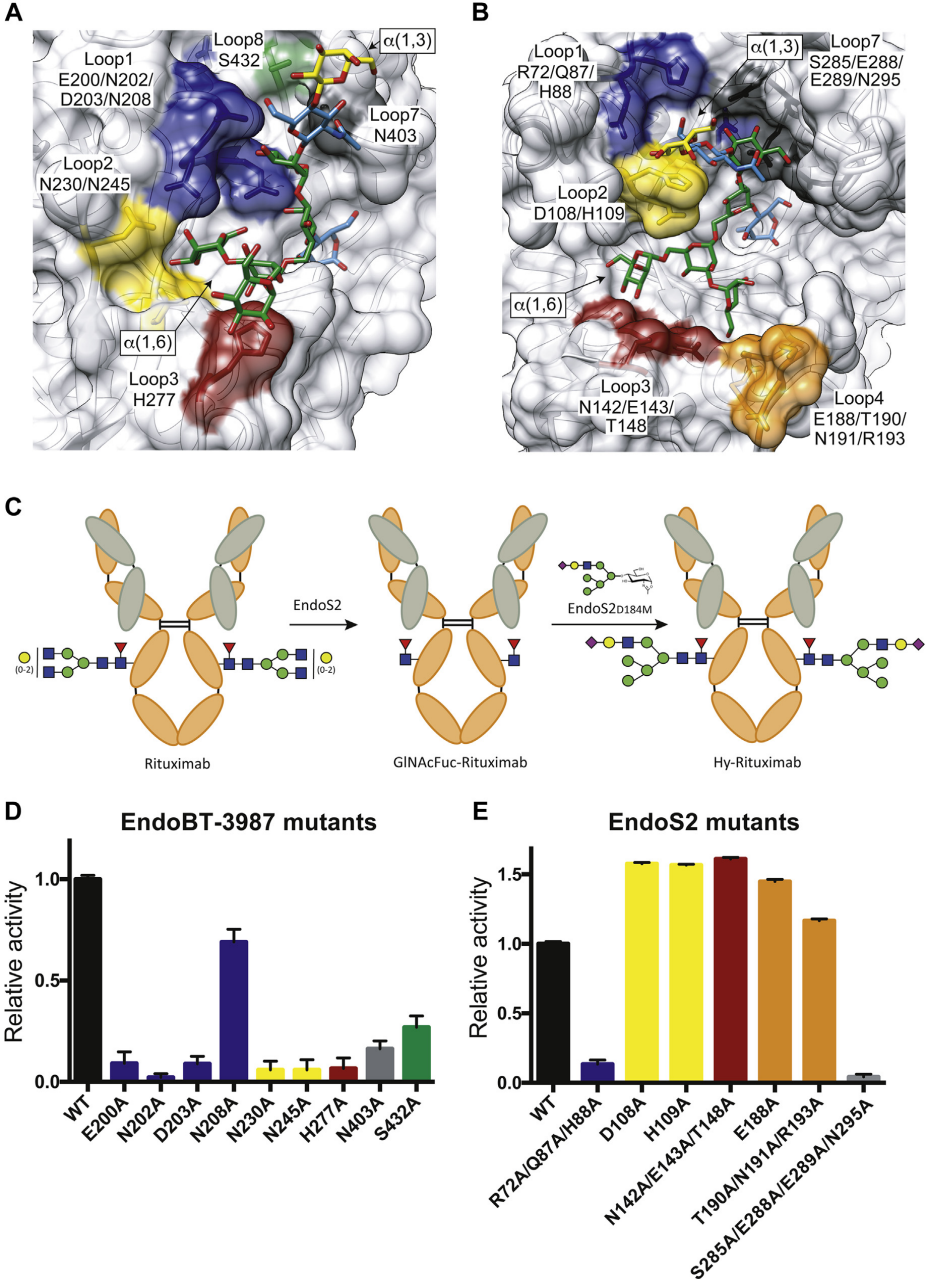
**Structural basis of Hy-type glycan recognition by EndoBT-3987 and EndoS2.***A*, surface representation of EndoBT-3987 in complex with the Hy-type glycan product, GalGlcNAcMan_5_GlcNAc, showing the alanine mutations performed in loop 1 (E200, N202, D203, or N208), loop 2 (N230 or N245), loop 3 (H277), loop 7 (N403), and loop 8 (S432). *B*, surface representation of EndoS2 in complex with the Hy-type glycan product showing the alanine mutations performed in loop 1 (R72, Q87, and H88), loop 2 (D108 or H109), loop 3 (N142, E143, and T148), loop 4 (E188 or T190, N191, and R193), and loop 7 (S285 and E288, E289, and N295) in the glycosidase domain. *C*, schematic representation of the chemoenzymatically remodeled Hy-rituximab. *D* and *E*, hydrolytic activity of EndoBT-3987 (*C*) and EndoS2 (*D*) wildtype and mutants against Hy-rituximab is shown, as determined by LC-MS analysis. Mutations on loops 1, 2, 3, 4, 7, and 8 are colored in *blue*, *yellow*, *red*, *orange*, *gray*, and *green*, respectively.

To further advance our understanding of Hy-type *N*-glycan recognition by GH18 ENGases we performed alanine scan mutagenesis of residues comprised in loops that decorate the *N*-glycan-binding sites of EndoBT-3987 and EndoS2 (Fig. 5, *A* and *B*). Rituximab is a chimeric monoclonal antibody approved for the treatment of B cell lymphoma. Commercial rituximab is composed primarily of CT-type *N*-glycans and small traces of Man_5_ and lacks Hy-type *N*-glycans (43). We will refer to it as CT-rituximab. To obtain a homogenously Hy-type *N*-glycan substrate, we chemoenzymatically synthesized Hy-rituximab from deglycosylated GlcNAcFuc-rituximab using the sugar oxazoline, SiaGalGlcNAc-Man_5_GlcNAc-ox, and EndoS2 glycosynthase mutant D184M, according to our previously reported method (44) (Fig. 5, *A*–*E*; Figs. S3 and S4; see Experimental procedures for details). Then, we determined the hydrolytic activities of these mutants against the synthesized Hy-rituximab by mass spectrometry. Specifically, we made alanine mutations of key residues in loop 1 (E200, N202, D203, or N208), loop 2 (N230 or N245), loop 3 (H277), loop 7 (N403), and loop 8 (S432) of EndoBT-3987; and loop 1 (R72, Q87, and H88), loop 2 (D108 or H109), loop 3 (N142, E143, and T148), loop 4 (E188 or T190, N191, and R193), and loop 7 (S285 and E288, E289 and N295) of EndoS2. The results reveal that mutations of the residues on loops 1, 2, 3, 7, and 8 of EndoBT-3987 critically reduced the enzymatic activity against Hy-type *N*-glycan, with the exception of N208, which does not interact with the Hy-type glycan in our crystal structure. The mutations of the residues on loops 1 and 7 of EndoS2 dramatically reduced the activity of the enzyme. This suggests that the interaction of EndoS2 with the glycan is mainly driven by the α(1,3) antenna of the Hy-rituximab substrate, since mutations of residues in these loops strongly reduced the enzymatic activity.

## Discussion

**Figure 6:**
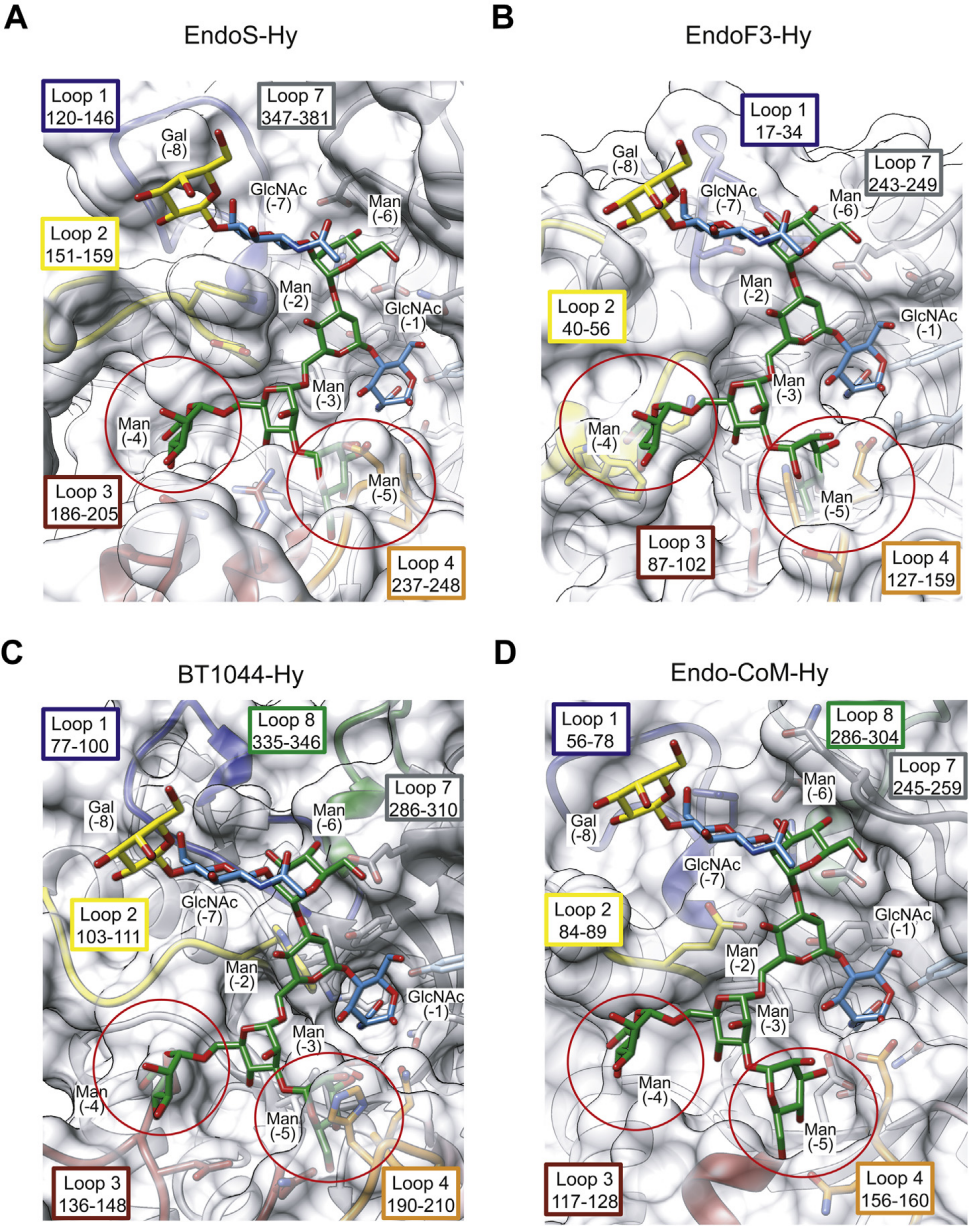
**Structural basis of EndoS, EndoF3, BT1044, and Endo-CoM specificity.** Molecular models of the docked Hy-type glycan product, GalGlcNAcMan_5_GlcNAc, in the binding site of (*A*) EndoS (PDB code 6EN3), (*B*) EndoF3 (PDB code 1EOM), (*C*) BT1044 (PDB code 6Q64), and (*D*) Endo-CoM (PDB code 6KPL).

The *N*-glycan specificity of the GH18 family ENGases is a matter of intense research. To date, three types of enzymes can be distinguished according to their *N*-glycan specificity: (i) enzymes that hydrolyze CT *N*-glycans but not HM-type *N*-glycans, (ii) enzymes that hydrolyze CT, HM-, and Hy-type *N*-glycans, and (iii) enzymes that hydrolyze HM- and Hy-type *N*-glycans but not CT-type *N*-glycans. The Hy-type *N*-glycan has a dual nature: its α(1,3) antenna is equivalent to the α(1,3) antenna of the CT-type *N*-glycans, whereas its α(1,6) antenna is covered into the α(1,6) antenna of HM-type *N*-glycans. Enzymes included in the first group, such as EndoS, EndoF3, BT1044 (37), and Endo-CoM (45) are unable to hydrolyze Hy-type *N*-glycans because the α(1,6) antenna is very bulky and the branched mannoses of this antenna cannot be allocated into the binding site due to steric hindrance with loops 2, 3, and 4 (Fig. 6) (46). Enzymes comprised in the second and third groups, such as EndoS2 and EndoBT-3987, respectively, are able to hydrolyze Hy-type *N*-glycans. We have previously performed alanine scanning mutagenesis followed by hydrolytic activity measurements with (i) EndoS2 using CT-rituximab, and HM-rituximab, obtained by treatment of HEK293 cells with kifunensine, inhibitor of the mannosidase I (47), as substrates, and (ii) EndoBT-3987 using HM-rituximab as substrate (39). Strikingly, the results revealed that EndoS2, in which a reduction of the enzymatic activity was observed against Hy-type *N*-glycans, showed the same behavior with CT-rituximab, supporting the notion that this enzyme predominantly recognizes the α(1,3) antenna. Conversely, even though interactions with the HM- and Hy-type *N*-glycans are mediated by the same residues in the crystal structures of EndoBT-3987-HM and EndoBT-3987-Hy, we observed in our hydrolytic experiments that the activity of the enzyme against Hy-rituximab was affected by mutations of all the residues that interact with the *N*-glycan. However, the activity of EndoBT-3987 against HM-rituximab was drastically modified by residues of loop 3 and to a lesser extent residues of loop 2 (Fig. S3). This could be explained by the low activity that EndoBT-3987 exhibited against Hy-rituximab and the different carbohydrate composition of both antennae of the Hy-type *N*-glycan compared with the HM-type. Specifically, Hy-type glycans lack both terminal mannoses on the α(1,6) antenna of Man9 that are present in HM-rituximab, in addition to the presence of the α(1,3) antenna that is similar to CT-type glycans.

**Figure 7:**
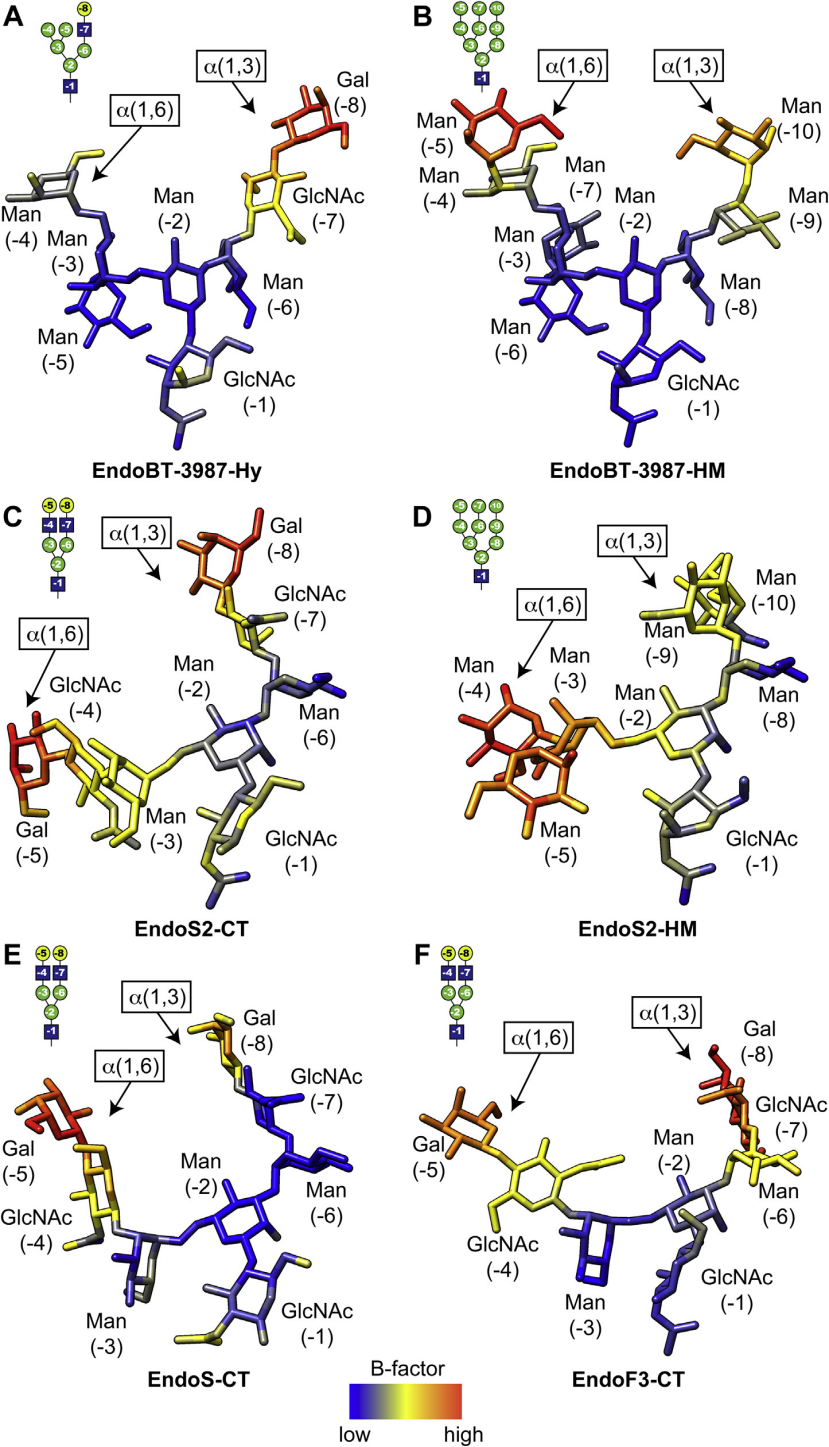
***N*-glycan flexibility on the active site of GH18 ENGases.** Relative B-factors of glycan products found in the crystal structures of EndoBT-3987-Hy (PDB code 7NWF) (*A*), EndoBT-3987-HM (PDB code 6T8L) (*B*), EndoS2-CT (PDB code 6MDS), (*C*) EndoS2-HM (PDB code 6MDV) (*D*), EndoS-CT (PDB code 6EN3) (*E*), and EndoF3 (PDB code 1EOM). The colors from *blue* to *yellow* and to *red* indicate B factors from small to large.

The interaction of ENGases with the GlcNAc (−1) is widely conserved and is mediated by hydrogen bonds and hydrophobic interactions contributing to the stabilization of the conformation of the intermediate of the enzymatic reaction. In the EndoBT-3987_WT_-Hy crystal structure, the α(1,3) antenna of the glycan interacts with loops 1 and 7, whereas the α(1,6) antenna interacts with loops 1, 2, 3, and 4 (Fig. 3). In our EndoS2-Hy molecular docking calculations, the α(1,3) antenna of the glycan interacts with loops 1, 2, and 7, whereas the α(1,6) antenna interacts with loops 2 and 3 (Fig. 4). Altogether, the experimental data indicate that EndoS2 and EndoBT-3987 recognize Hy-type *N*-glycans following two different mechanisms. Hy-type *N*-glycan recognition by EndoS2 is mainly mediated by the interaction of the enzyme with the α(1,3) antenna of the *N*-glycan and to a lesser extent with the α(1,6) antenna. In contrast, the interaction of EndoBT-3987 with Hy-type *N*-glycans is driven by the α(1,6) antenna. B-factors analysis of the individual carbohydrate residues in the X-ray crystal structures of GH18 ENGases in complex with glycan products (Fig. 7) revealed that the smallest B-factors belong to the carbohydrate residues that form the core and the α(1,6) antenna in the case of EndoBT-3987 crystal structures regardless of whether it is HM-type or Hy-type glycans (Fig. 7, *A* and *B*). These smallest B-factors suggest a lower flexibility of these carbohydrate residues owing to a stable interaction with the protein. On the contrary, the smallest B-factors were found in carbohydrates that form the core and α(1,3) antenna in the structures of EndoS2 in complex with HM-type and CT-type glycans (Fig. 7, *C* and *D*) and EndoS in complex with CT-type glycans (Fig. 7*E*). In addition, the B-factors of carbohydrates of α(1,6) antenna are smaller than those of α(1,3) in the structure of EndoF3 because this enzyme also hydrolyzes triantennary CT glycans, which requires some flexibility of this antenna in order to accommodate larger glycans at the binding site.

**Figure 8:**
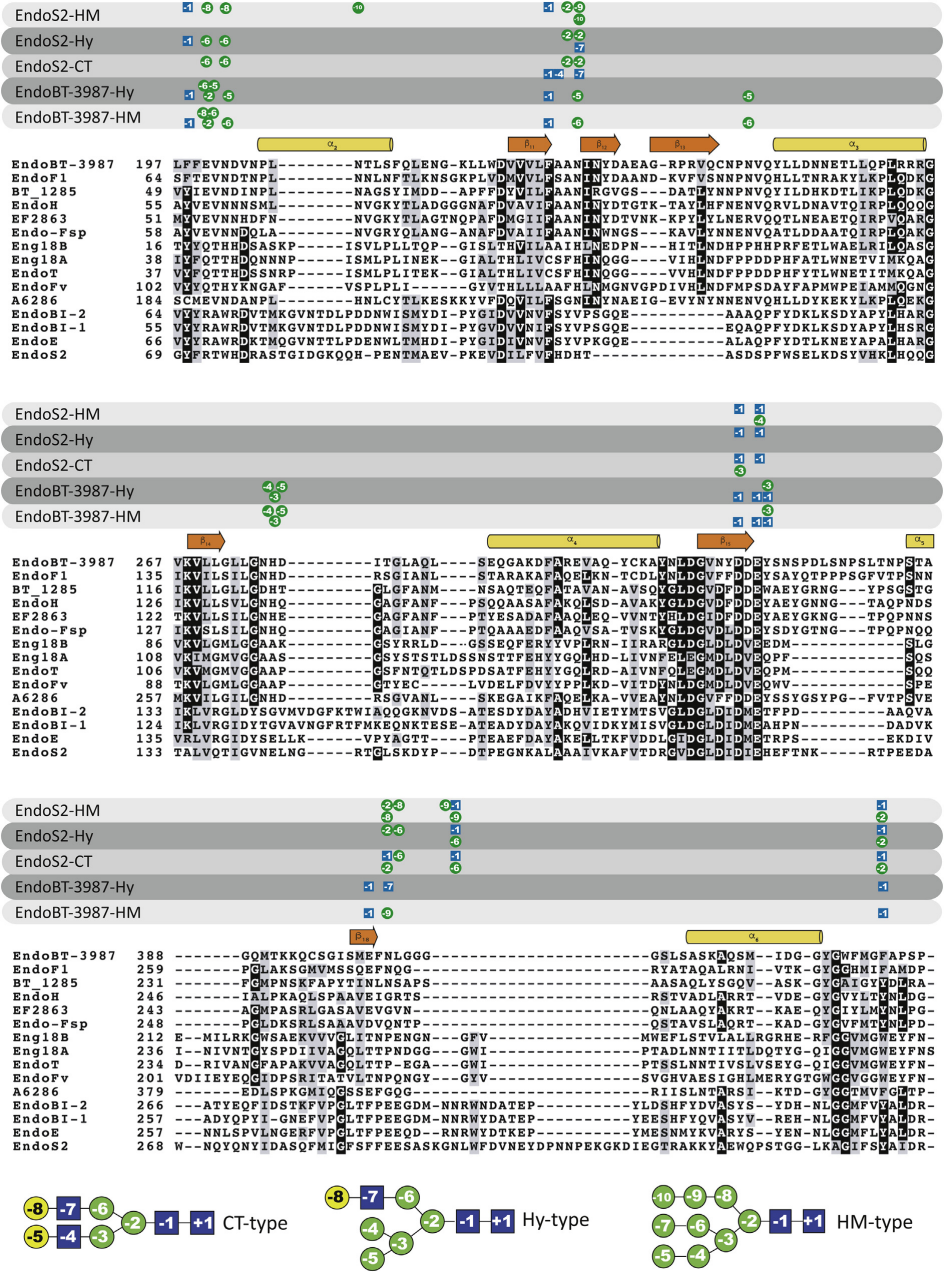
**Structure-based sequence alignment of EndoS2 and EndoBT-3984 with GH18 family enzymes with known HM-type *N*-glycan specificity.** Comparison of BT3987 from *B. thetaiotaomicron* VPI-5482 (Q8A0N4, Uniprot code), EndoF1 from *Elizabethkingia meningoseptica* (P36911, Uniprot code), BT1285 from *B. thetaiotaomicron* VPI-5482 (Q8A889, Uniprot code), EndoH from *Streptomyces plicatus* (P04067, Uniprot code), EF2863 from *Enterococcus faecalis*(Q830C5, Uniprot code), Endo-Fsp from *Flavobacterium* sp. (P80036, Uniprot code), Eng18B from *Hypocrea atroviride IMI 206040* (G9P8KO, Uniprot code), Eng18A from *Hypocrea atroviride IMI 206040* (G9NR36, Uniprot code), EndoT from *Hypocrea jecorina* (C4RA89, Uniprot code), EndoFv from *Flammulina velutipe*s (D1GA49, Uniprot code), A6286 from *Prevotella melaninogenica* (D9RSV7, Uniprot code), EndoBI-2 from *Bifidobacterium longum* subsp. *infantis*, (E8MUK6, Uniprot code), EndoBI-1 from *Bifidobacterium longum* subsp. *infantis*, (B7GPC7, Uniprot code), and EndoE and EndoS2 from *Streptococcus pyogenes* (T1WGN1, Uniprot code). Residues that interact with a specific carbohydrate of the *N*-glycan in the crystal structure of EndoS2-HM (PDB code 6MDV), EndoS2-CT (PDB code 6MDS), EndoBT-3987-HM (PDB code 6TK8), and EndoBT-3987-Hy (PDB code 7NWF) and the molecular docking calculations of Hy into the EndoS2-binding site are highlighted with the carbohydrate symbol and number.

**Figure 9:**
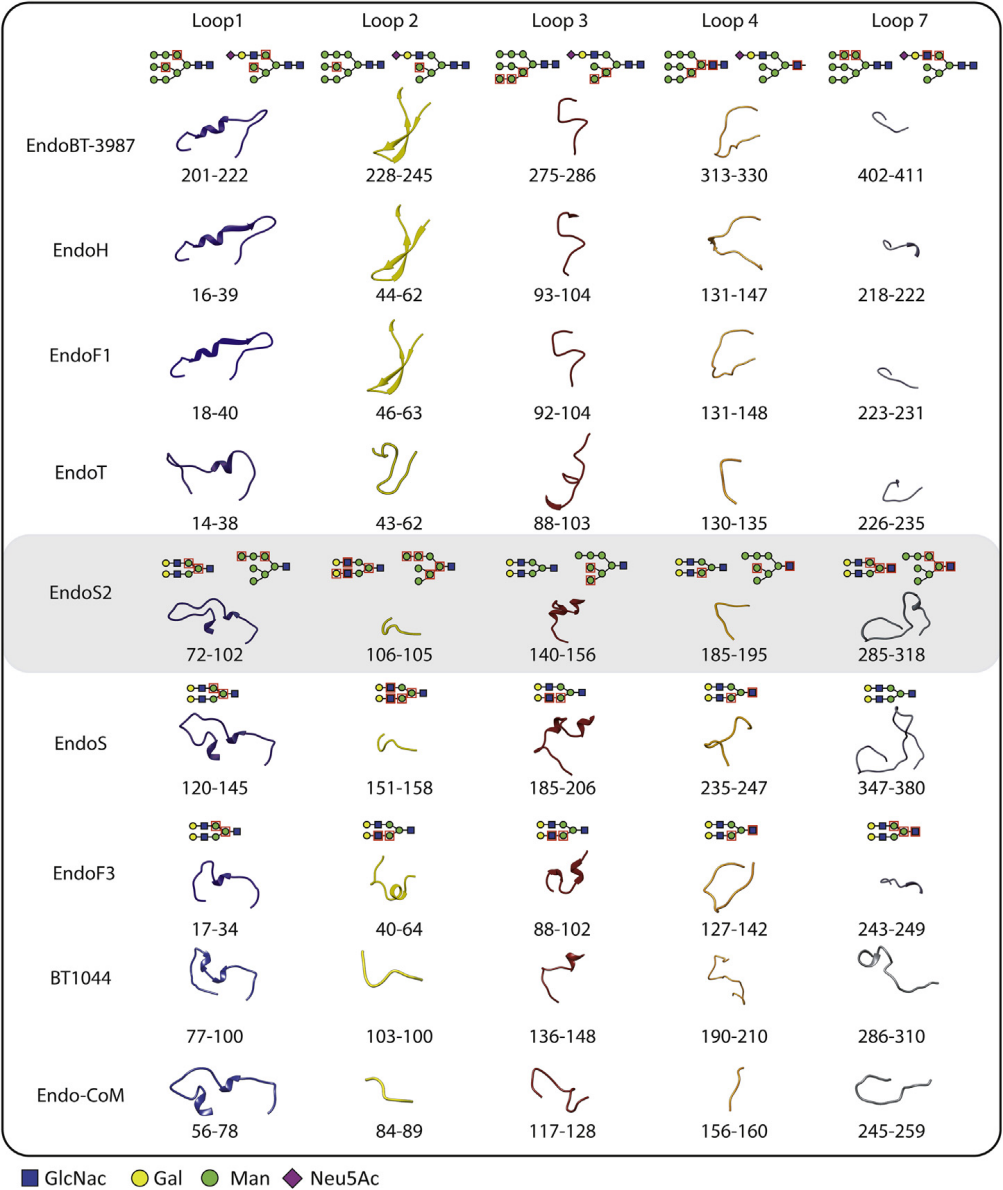
**Structural basis of EndoS2 and EndoBT-3987 specificity for Hy-type *N*-glycans.** Structural comparison of the loops surrounding the active site of GH18 family enzymes with ENGase activity and known X-ray crystal structure: EndoBT-3987 (PDB codes 6T8K and 7NWF), EndoH (PDB code 1C3F), EndoF1 (PDB code 2EBN), EndoT (PDB code 4AC1), EndoS2 (PDB codes 6MDS and 6MDV), EndoS (PDB code 6EN3), EndoF3 (1EOM), BT1044 (PDB code 6Q64), and Endo-CoM (6KPN). Carbohydrate moieties that interact with each loop in the crystal structures are marked with *red squares*.

Moreover, the structure-based sequence alignment of EndoS2 and EndoBT-3984 with GH18 family enzymes with known HM-type *N*-glycan specificity shows the conservation of residues that interact with the *N*-glycans in the GH18 ENGase family, suggesting that the enzymes that hydrolyze CT-type, HM-type, and Hy-type recognize mainly the glycan by the α(1,3) antenna while the enzymes that hydrolyze HM-type and Hy-type glycans recognize the α(1,6) antenna (Fig. 8). In this alignment, we can distinguish two groups based on the high sequence homology of the *N*-glycan interacting loops. One group is composed of EndoBT-3987, EndoF1, BT_1285, EndoH, EF2863, Endo-Fsp, Eng18B, Eng18A, EndoT, EndoFv, and A6286, and a second group is composed of EndoS2, EndoE, EndoBI-2, and EndoBI-1 (Fig. 8). The structural comparison of the loops surrounding the active site of the GH18 family of ENGases support the basis for the CT and HM-type recognition mechanisms of this group of enzymes (Fig. 9). We established two groups of GH18 enzymes based on their loop conformations: enzymes that hydrolyze CT-type *versus* HM-type glycans, with the exception of EndoS2 showing subtle differences in the glycoside hydrolase domain structure that allows the enzyme to recognize CT, Hy-type, and HM-type glycans following a similar mechanism (Fig. 9) (47). Our structural analysis revealed significant loop conformation similarities between enzymes that hydrolyze CT-type or HM-type glycans; however, no loop conformation similarities were found between enzymes that hydrolyze Hy-type *N*-glycan (Fig. 9). Most ENGases have evolved to process more than a single *N*-glycan, although they have different hydrolytic efficiencies against distinct *N*-glycan substrates. The dual nature of Hy-type *N*-glycans with a CT α(1,3) antenna and a HM-type α(1,6) antenna allows this glycan to be recognized as a CT (*e.g.*, EndoS2) or as a HM-type substrate (*e.g.*, EndoBT-3987, EndoF1, EndoH) by ENGases of family GH18, suggesting that each different group of enzymes based on their glycan specificity recognizes several *N*-glycans following a common mechanism.

It is worth noting that *Bacteroides* is one of the predominant genera of the human gut (48). Thousands of different CAZymes are encoded in Bacteroidetes genome in order to facilitate the degradation of glycans from the host itself or its diet (49, 50, 51). Polysaccharide utilization loci (PULs) (52) are discrete clusters that encode groups of enzymes and glycan-binding proteins that typically orchestrate the degradation of a specific glycan in the human gut. In that context, the identification of enzymes that orchestrate the hydrolysis of Hy-type *N*-glycans in the human gut remains unknown. In *B. thetaiotaomicron*, the first step in the degradation of HM-type and CT *N*-glycans is mediated by the action of two GH18 ENGases, EndoBT-3987 and BT1044, respectively (37, 39). EndoBT-3987 belongs to PUL-72, whereas BT1044 belongs to PUL 13 (36, 37). Of interest, BT1044 only hydrolyzes CT *N*-glycans and not HM- or Hy-type *N*-glycans (37). The closest structural homologue of BT1044 in the GH18 ENGase family is EndoF3, a CT-type-specific processing enzyme (37). Here, we show that EndoBT-3987 displays hydrolytic activity against Hy-type *N*-glycans, strongly suggesting that this enzyme could initiate and/or participate in the degradation of this type of glycan in the human gut. We propose that once the Hy-type *N*-glycans are hydrolyzed in the extracellular environment by EndoBT-3987, they could be introduced into the periplasm by the Sus-like (36, 53, 54) porins and accessory proteins from the HM and/or CT degradation PULs (55, 56). Glycoside hydrolases that belong to the HM-type degradation PUL would degrade the α(1,6) antenna of the Hy-type *N*-glycan, whereas glycoside hydrolases that belong to PULs involved in the degradation of CT-type *N*-glycans could hydrolyze the α(1,3) antenna.

## Conclusions

In summary, we have determined two independent mechanisms for recognition of Hy-type *N*-glycans by paradigmatic members of the GH18 family of ENGases, EndoBT-3987 and EndoS2. EndoBT-3987 and EndoS2 hydrolyze Hy-type *N*-glycans recognizing the α(1,6) and α(1,3) antenna, respectively. Both enzymes play important biological functions. EndoS2 is secreted by *S. pyogenes* serogroup M49 that allows the bacterium to remove more than 20 glycoforms from antibodies, eliminating their effector functions to evade the immune system. EndoBT-3987 from *B. thetaiotaomicron* has been described as the enzyme that initiates the HM-type *N*-glycan processing in the human gut. Our *in vitro* experiments show that this enzyme might also be responsible for the Hy-type *N*-glycan processing in host mucins (57).

ENGases are versatile enzymes for biotechnological applications, including glycan analysis and glycosylation remodeling of heterogeneous glycoproteins. EndoS2 glycosynthase mutants from *S. pyogenes* are key tools to glycoengineer immunotherapeutic IgG monoclonal antibodies. Because of the broad *N*-glycan specificity of EndoS2, this enzyme is able to hydrolyze, and as a glycosynthase mutant to generate, a more diverse set of *N*-glycans on antibodies (44, 47). In addition, the use of EndoBT-3987 and EndoS2 would allow introduction of structural variability as glycosynthases, in the α(1,3) and α(1,6) antennae, respectively. The detailed knowledge of the ENGases substrate recognition mechanism is critically important for rationalizing the glycoengineering of glycoproteins, which will positively impact the treatment and diagnosis of myriad human diseases.

## Experimental procedures

### Materials

EndoS2 and EndoBT-3987 wildtype and the corresponding mutants were expressed and purified to apparent homogeneity as described (39, 47). Monoclonal antibody rituximab was purchased from Premium Health Services Inc.

### EndoBT-3987_WT_-Hy complex crystallization and data collection

The EndoBT-3987_WT_-Hy complex was crystallized by mixing 0.25 μl of a protein solution at 10 mg ml^−1^ in 20 mM Tris-HCl pH 7.5, 50 mM NaCl, and 2.5 mM of the oligosaccharide Neu_5_AcGalGlcNAcMan_5_GlcNAc, with 0.25 μl of 100 mM Bis-Tris propane pH 6.0, 200 mM NaF, and 20% (w/v) PEG 3350. Crystals grew in 1 to 2 days. They were transferred to a cryoprotectant solution containing 10% glycerol and frozen under liquid nitrogen. Complete X-ray diffraction datasets were collected at beamline I24 (Diamond Light source). Datasets were integrated and scaled with X-ray Detector Software (XDS) following standard procedures (58). The EndoBT-3987_WT_-Hy complex crystallized in the orthorhombic space group *P* 2_1_ 2_1_ 2_1_ with one molecule in the asymmetric unit and diffracted to a maximum resolution of 2.0 Å.

### EndoBT-3987_WT_-Hy structure determination and refinement

The EndoBT-3987_WT_-Hy complex structure was solved by molecular replacement methods, using the PDB code 6T8I as a template, implemented in Phaser (59) and the PHENIX suite (60). Model rebuilding was carried out with Buccaneer (61) and the *CCP4* suite (62). The final manual building was performed with Coot (63) and refinement with phenix.refine (64). The structure was validated by MolProbity (65). Data collection and refinement statistics are presented in Table S1. Atomic coordinates and structure factors have been deposited with the Protein Data Bank, accession codes 7NWF. Molecular graphics and structural analyses were performed with the UCSF Chimera package (66).

### Chemoenzymatic synthesis of Hy N-glycans

The Hy-type *N*-glycan was prepared through a chemoenzymatic method by using truncated HM-type *N*-glycan as the starting material. The HM-type *N*-glycan was isolated from soybean flour and then truncated into the Man_5_ *N*-glycoform as reported (39). Man_5_-Asn was first labeled with the fluorenylmethoxycarbonyl group (Fmoc) to facilitate the purification in each step. Briefly, GlcNAcMan_5_GlcNAc_2_-AsnFmoc was synthesized by transferring a GlcNAc moiety to a mannose unit of Man_5_-Asn at the α(1,3)-antenna using the human β1,2-*N*-acetylglucosaminyltransferase I, GnTI (MGATI) (67). Then GalGlcNAcMan_5_GlcNAc_2_-AsnFmoc was prepared using a β1,4-Gal-T from *Neisseria meningitidis* (44). The resulting Gal modified product was sialylated through a one-pot two-enzyme reaction system with a α2,6-sialyltransferase from *Photobacterium damselae* (68) (Pd26ST) and CMP-sialic acid synthetase from *N. meningitidis* (CSS) to afford the SiaGalGlcNAcMan_5_GlcNAc_2_-AsnFmoc. Finally, the aglycone portion, AsnFmoc, and the first GlcNAc were cleaved off together by an endoglycosidase, EndoS2, and the desired product was purified using a Sephadex G-15 size-exclusion column (GE Healthcare) to afford pure SiaGalGlcNAc-Man_5_GlcNAc *N*-glycan as a white powder after lyophilization. The pure Hy-type *N*-glycan was characterized with electrospray ionization mass spectrometry (ESI-MS). ESI-MS: calcd for Hyb-GlcNAc, *M* = 1688.74 Da; found (*m/z*), 845.39 [M + 2H]^2+^, 1689.78 [M + H]^+^.

### Chemoenzymatic remodeling of rituximab N-glycans

The hybrid-type *N*-glycan oxazoline substrate, deglycosylated antibody, and the Hy-type *N*-glycoform of antibody were prepared according to our previously reported method (44). Briefly, sugar oxazoline (3.3 mg, 60 equiv.), SiaGalGlcNAc-Man_5_GlcNAc-ox, was added into a solution of deglycosylated GlcNAcFuc-rituximab (5 mg) and EndoS2 glycosynthase mutant D184M (0.1 mg ml^−1^) in a buffer (PBS, pH 7.4, 150 mM) at 30 °C for 1 h. After the completion of the transglycosylation reaction, the glycoengineered antibody was purified with Protein A affinity chromatography and characterized with ESI-MS.

### LC-MS enzymatic activity assays of EndoBT-3987 and EndoS2 wildtype and alanine mutants

Reactions for the EndoS2 alanine scan mutants were set up using 5 nM EndoS2 with 5 μM Hy-rituximab substrate, in PBS pH 7.4 at room temperature. Ten-microliter reactions were set up in triplicate and allowed to proceed for 60 min before being quenched with 1.1 μl of 1% trifluoroacetic acid. For the EndoBT-3987 alanine scan, 20-μl reactions were set up in triplicate using 1 μM EndoBT-3987 and 100 nM Hy-rituximab substrate in PBS pH 7.4 at room temperature. The reactions were sampled after 24 h. The reactions were analyzed by LC-MS using an Agilent 1290 Infinity II LC System equipped with a 50-mm PLRP-S column from Agilent with 1000-Å pore size. The LC system is attached to an Agilent 6560 Ion Mobility (IM) quadrupole time-of-flight (Q-TOF) mass spectrometer (Agilent). Relative amounts of the substrate and hydrolysis products were quantified after deconvolution of the raw data, and the corresponding peaks were identified using BioConfirm (Agilent). The data were plotted and statistical significance was determined using a multiple comparisons test (Tukey method) in GraphPad (GraphPad Software).

### Molecular docking calculations

The Hy product (Gal_1_GlcNAc_1_Man_3_GlcNAc_1_) was modeled using GLYCAM-Web website (Complex Carbohydrate Research Center, University of Georgia; http://www.glycam.com). Ligand docking was performed using AutoDock Vina employing standard parameters (69).

## Data availability

The atomic coordinates of the EndoBT-3987-Hy complex have been deposited in the Protein Data Bank, PDB ID 7NWF (www.rcsb.org).

## Supporting information

This article contains supporting information (39, 47).

## Conflict of interest

The authors declare that they have no conflicts of interest with the contents of this article.
