# Supplementary material for: GH18 endo-β-N-acetylglucosaminidases use distinct mechanisms to process hybrid-type N-linked glycans
Source: J Biol Chem. 2021 Jul 26;297(2):101011. doi: 10.1016/j.jbc.2021.101011 (PMC8374693; doi:10.1016/j.jbc.2021.101011)
Supplement: Supplemental Figures S1–S4 and Table S1 [file mmc1.pdf]

SUPPLEMENTAL INFORMATION

GH18 ENDO-β-*N*-ACETYLGLUCOSAMINIDASES USE DISTINCT MECHANISMS TO PROCESS HYBRID-TYPE *N*-LINKED GLYCANS

Beatriz Trastoy,[1,2,†,*] Jonathan J. Du,[3,†] Chao Li,[4] Mikel García-Alija,[1,2] Erik H. Klontz,[5,6] Blaine R. Roberts,[3] Thomas C. Donahue,[4] Lai-Xi Wang,[4] Eric J. Sundberg,[3,*] and Marcelo E. Guerin[1,2,7,*]

[1] Structural Glycobiology Lab, Structural Biology Unit, Center for Cooperative Research in Biosciences (CIC bioGUNE), Basque Research and Technology Alliance (BRTA), Bizkaia, Technology Park, Building 801A, 48160 Derio, Spain
[2] Structural Glycobiology Lab, IIS-BioCruces Bizkaia Cruces Plaza, 48903 Barakaldo, Bizkaia, Spain.
[3] Department of Biochemistry, Emory University School of Medicine, Atlanta, GA 30322, USA.
[4] Department of Chemistry and Biochemistry, University of Maryland, College Park, MD 20742, USA
[5] Institute of Human Virology, University of Maryland School of Medicine, Baltimore, MD 21201, USA
[6] Department of Microbiology and Immunology, University of Maryland School of Medicine, Baltimore, MD 21201, USA
[7] Ikerbasque, Basque Foundation for Science, 48009 Bilbao, Spain

Running title: *N-glycan processing by endo-β-N-acetylglucosaminidases*

*To whom correspondence should be addressed: Beatriz Trastoy, Structural Glycobiology Laboratory, IIS-BioCruces Bizkaia Cruces Plaza, 48903 Barakaldo, Bizkaia, Spain, beatriz.trastoy@gmail.com; Eric J. Sundberg, Department of Biochemistry, Emory University School of Medicine, Atlanta, GA 30322, USA, eric.sundberg@emory.edu; Marcelo E. Guerin, Structural Glycobiology Laboratory, IIS-BioCruces Bizkaia Cruces Plaza, 48903 Barakaldo, Bizkaia, Spain, mrcguerin@gmail.com.

[†] These authors contributed equally

TABLE OF CONTENTS

1. **SUPPLEMENTAL TABLES**

   Table S1. Data collection and refinement statistics.

2. **SUPPLEMENTAL FIGURES**

   Figure S1. Electron density maps of the refined EndoBT-3987 complex in the presence of GlcNAcMan$_5$GlcNAc.

   Figure S2. The catalytic mechanism of EndoBT-3987.

   Figure S3. Biochemical synthesis of Neu$_5$AcGalGlcNAcMan$_5$GlcNAc.

   Figure S4. Comparison of hydrolytic activity of EndoBT-3987 and EndoS2 against Rituximab with defined glycoforms.

3. **SUPPLEMENTAL REFERENCES**

1. SUPPLEMENTAL TABLES

Table S1. Data collection and refinement statistics.

	**EndoBT-3987$_{WT}$-Hy**
PDB code<br>Beamline	7NWF<br>I24 (DLS) 6/04/2019
Wavelength (Å)	0.9792
Resolution range (Å)	45.9 - 2.0 (2.07 - 2.0)
Space group	P 21 21 21
Unit cell	49.29, 74.02, 125.54, 90, 90, 90
Total reflections	195095 (15200)
Unique reflections	30248 (2392)
Multiplicity	6.4 (6.4)
Completeness (%)	95.06 (76.87)
Mean I/sigma(I)	12.58 (2.57)
Wilson B-factor	24.51
R-merge	0.10 (0.63)
R-meas	0.11 (0.68)
CC1/2	0.99 (0.85)
CC*	0.99 (0.96)
Reflections used in refinement	30241 (2389)
Reflections used for R-free	1483 (114)
R-work	0.18 (0.23)
R-free	0.21 (0.27)
CC(work)	0.96 (0.89)
CC(free)	0.95 (0.77)
Number of non-hydrogen atoms	3711
macromolecules	3367
ligands	107
Protein residues	432
RMS(bonds)	0.013
RMS(angles)	1.23
Ramachandran favored (%)	97.44
Ramachandran allowed (%)	2.56
Ramachandran outliers (%)	0.00
Rotamer outliers (%)	0.82
Clashscore	3.08
Average B-factor	25.65
macromolecules	25.35
ligands	32.24
solvent	26.84

Statistics for the highest-resolution shell are shown in parentheses

2. SUPPLEMENTAL FIGURES

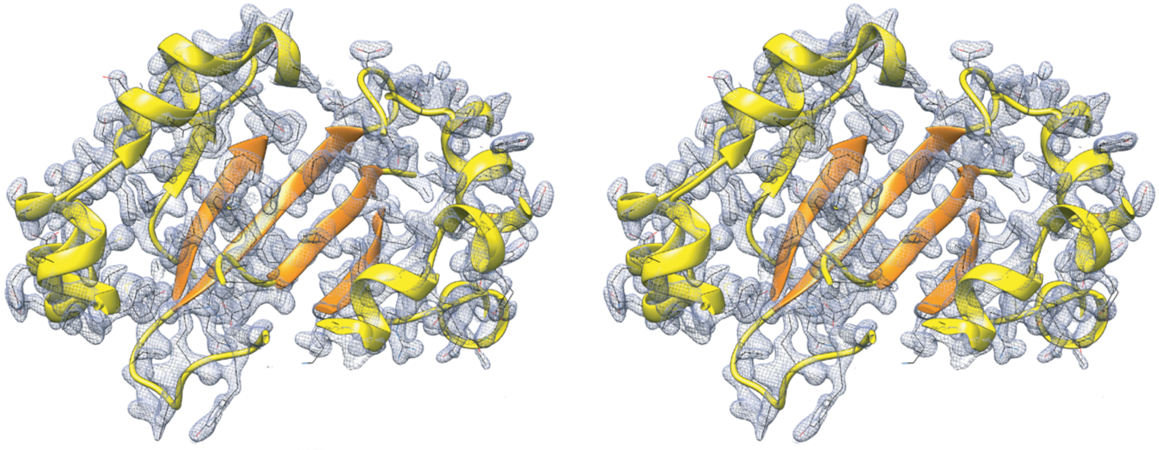

Figure S1. Electron density maps of the refined EndoBT-3987 complex in the presence of GalGlcNAcMan$_5$GlcNAc. Stereo view of the final electron density maps (2mFo-DFc contoured at 1σ) corresponding to the EndoBT-3987in complex with the Hy-type *N*-glycan.

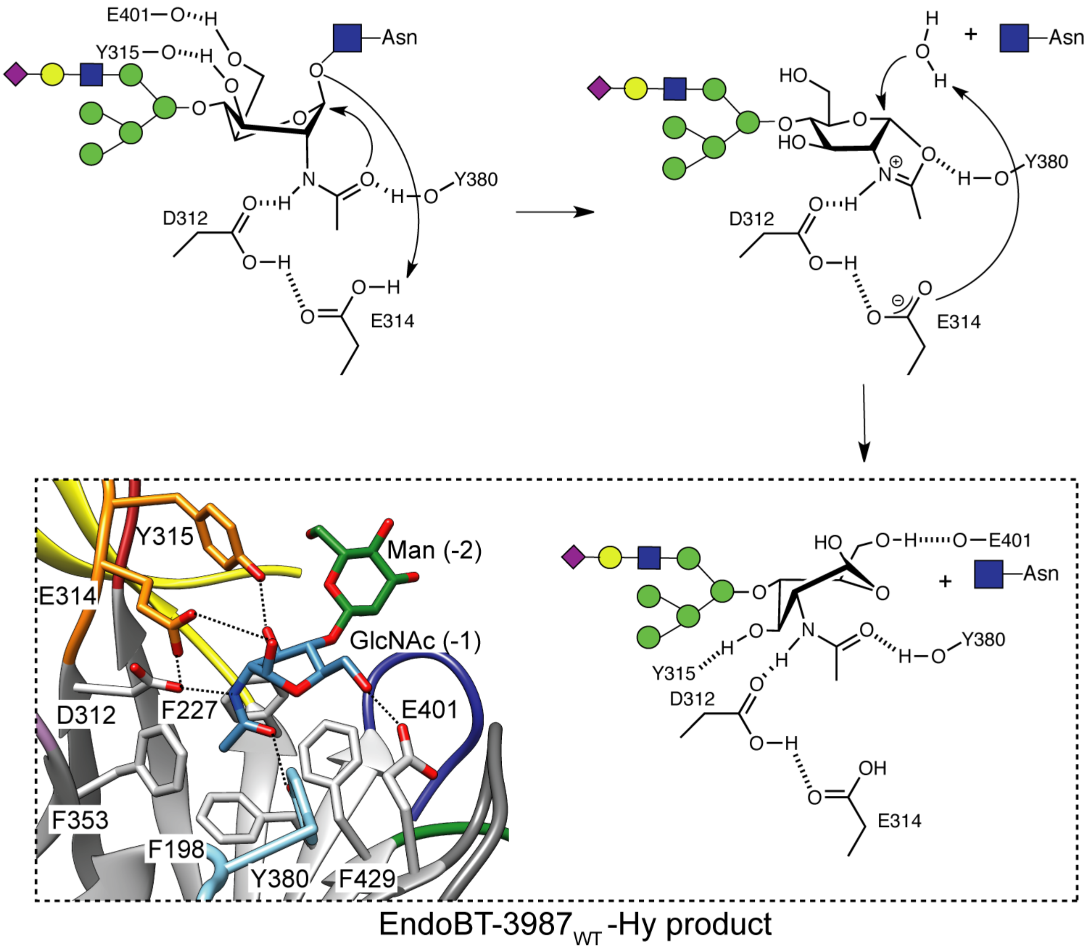

Figure S2. The catalytic mechanism of EndoBT-3987. In the first step, the *N*-linked Hy-type glycan substrate binds to the active site inducing the distortion of the GlcNAc (-1). In this step, the E314 residue protonates the glycosidic bond, acting as an acid, whereas the D312 residue orients the oxygen of the C2-acetamide group of GlcNAc (-1), which attacks the anomeric carbon of GlcNAc (-1) and leads to the formation of an oxazolinium intermediate. In the second step, E314 acts as a base, deprotonating a water molecule that performs a second nucleophilic attack and breaks the oxazoline ring, regenerating the hemiacetal sugar with retention of anomeric configuration.

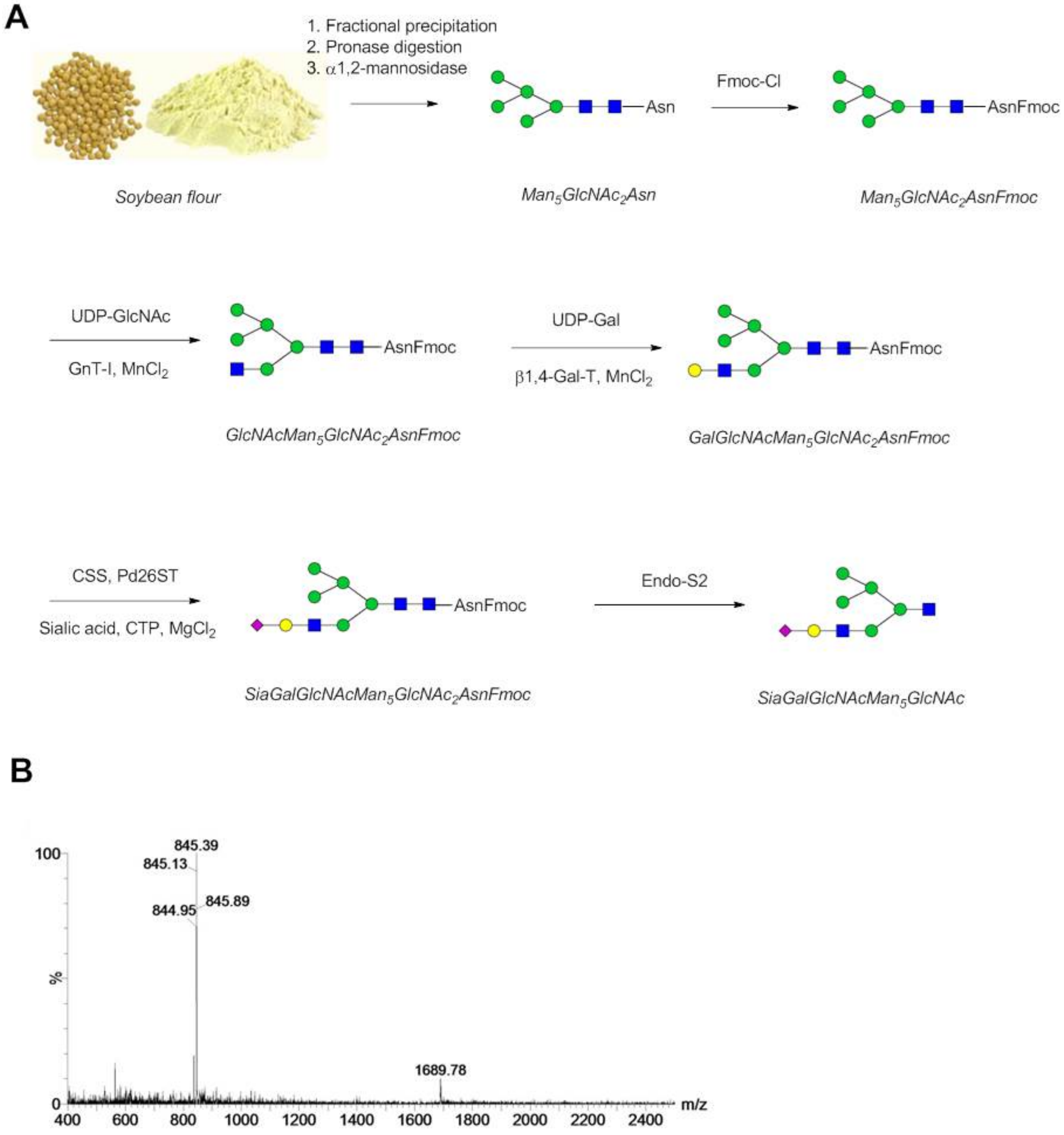

Figure S3. Biochemical synthesis of $Neu_5AcGalGlcNAcMan_5GlcNAc$. (A) Chemoenzymatic synthesis of Hy-type *N*-glycan. (B) ESI-MS spectrum of the synthetic Hy-type *N*-glycan.

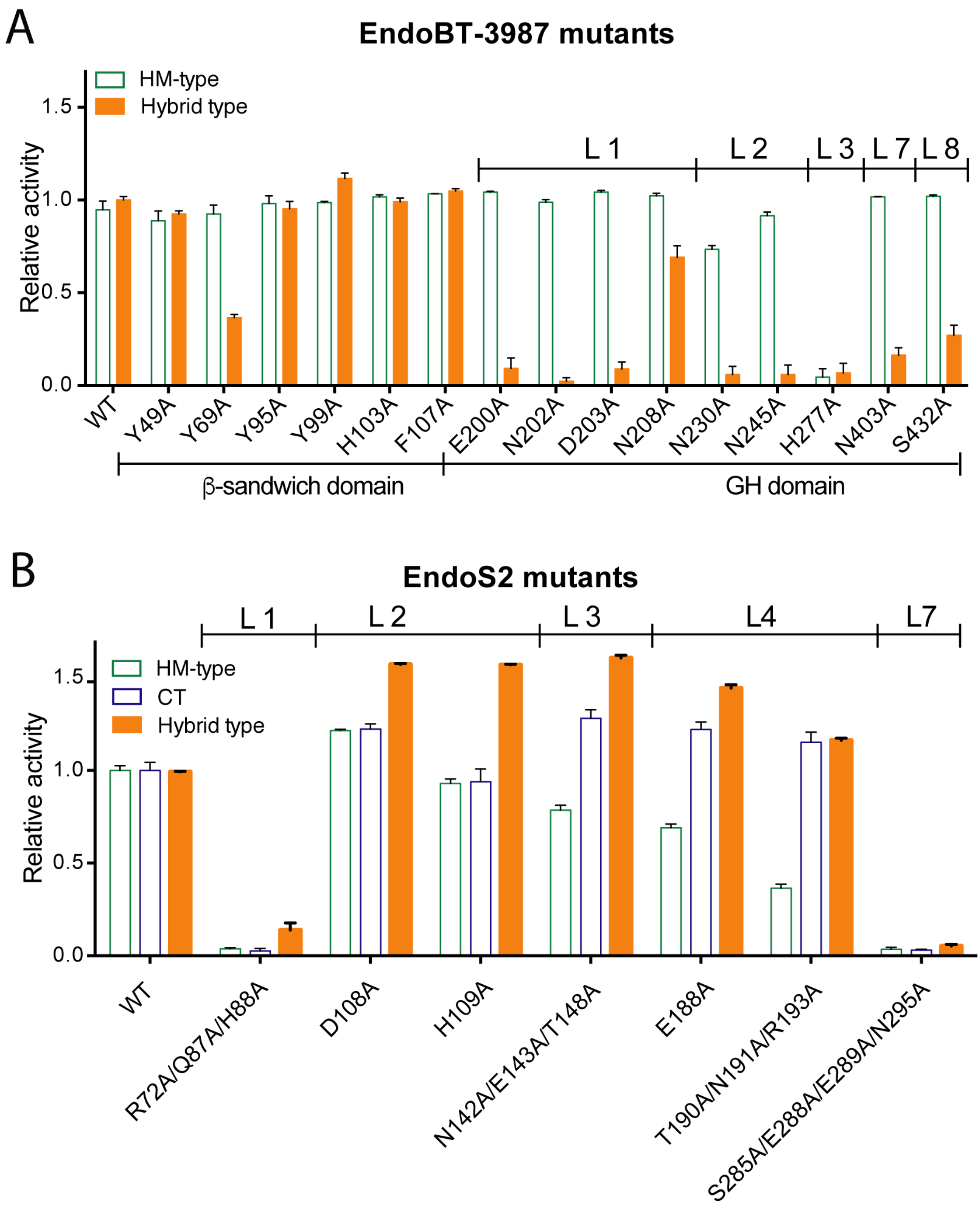

Figure S4. Comparison of hydrolytic activity of EndoBT-3987 and EndoS2 against Rituximab with defined glycoforms. (A) Hydrolytic activity of EndoBT-3987 and mutants determined by LC-MS analysis against HM-Rituximab (white and green bars) previously described[1] and Hy-Rituximab (orange bars) performed in this study. (B) Hydrolytic activity of EndoS2 and mutants determined by LC-MS analysis against HM-Rituximab (white and green bars) and Rituximab (white and blue bars) previously described[2] and Hy-Rituximab (orange bars) performed in this study. The hydrolytic activity data is normalized against the activity of EndoBT-3987 and EndoS2 wild type against each Rituximab substrate.

3. SUPPLEMENTAL REFERENCES

(1) Trastoy, B.; Du, J. J.; Klontz, E. H.; Li, C.; Cifuente, J. O.; Wang, L. X.; Sundberg, E. J.; Guerin, M. E. Structural Basis of Mammalian High-Mannose N-Glycan Processing by Human Gut Bacteroides. *Nat. Commun.* **2020**, *11* (1), 899.

(2) Klontz, E. H.; Trastoy, B.; Deredge, D.; Fields, J. K.; Li, C.; Orwenyo, J.; Marina, A.; Beadenkopf, R.; Günther, S.; Flores, J.; Wintrode, P. L.; Wang, L. X.; Guerin, M. E.; Sundberg, E. J. Molecular Basis of Broad Spectrum N-Glycan Specificity and Processing of Therapeutic IgG Monoclonal Antibodies by Endoglycosidase S2. *ACS Cent. Sci.* **2019**, *5* (3), 524–538.
